# Textile Dye Removal from Wastewater Effluents Using Bioflocculants Produced by Indigenous Bacterial Isolates 

**DOI:** 10.3390/molecules171214260

**Published:** 2012-11-30

**Authors:** Simphiwe P. Buthelezi, Ademola O. Olaniran, Balakrishna Pillay

**Affiliations:** Department of Microbiology, School of Life Sciences, College of Agriculture, Engineering and Science, University of KwaZulu-Natal (Westville Campus), Private Bag X54001, Durban 4000, South Africa; E-Mails: buthelezisim@ukzn.ac.za (S.P.B.); pillayb1@ukzn.ac.za (B.P.)

**Keywords:** bioflocculant, decolourization, dyes, flocculation, wastewater effluents

## Abstract

Bioflocculant-producing bacteria were isolated from activated sludge of a wastewater treatment plant located in Durban, South Africa, and identified using standard biochemical tests as well as the analysis of their 16S rRNA gene sequences. The bioflocculants produced by these organisms were ethanol precipitated, purified using 2% (w/v) cetylpyridinium chloride solution and evaluated for removal of wastewater dyes under different pH, temperature and nutritional conditions. Bioflocculants from these indigenous bacteria were very effective for decolourizing the different dyes tested in this study, with a removal rate of up to 97.04%. The decolourization efficiency was largely influenced by the type of dye, pH, temperature, and flocculant concentration. A pH of 7 was found to be optimum for the removal of both whale and mediblue dyes, while the optimum pH for fawn and mixed dye removal was found to be between 9 and 10. Optimum temperature for whale and mediblue dye removal was 35 °C, and that for fawn and mixed dye varied between 40–45 °C and 35–40 °C, respectively. These bacterial bioflocculants may provide an economical and cleaner alternative to replace or supplement present treatment processes for the removal of dyes from wastewater effluents, since they are biodegradable and easily sustainable.

## 1. Introduction

The use of synthetic chemical dyes in various industrial processes, including paper and pulp manufacturing, plastics, dyeing of cloth, leather treatment and printing has increased considerably over the last few years, resulting in the release of dye-containing industrial effluents into the soil and aquatic ecosystems [1]. Since most of these dyes are toxic in nature, their presence in industrial effluents is of major environmental concern because they are usually very recalcitrant to microbial degradation [2]. In some cases, the dye solution can also undergo anaerobic degradation to form potentially carcinogenic compounds that can end up in the food chain [3]. Moreover, highly coloured wastewaters can block the penetration of sunlight and oxygen, essential for the survival of various aquatic forms [4]

Textile industries utilize substantial volumes of water and chemicals for wet-processing of textiles. These chemicals, ranging from inorganic compounds and elements to polymers and organic products are used for desiring, scouring, bleaching, dyeing, printing, and finishing [5]. There are more than 8,000 chemical products associated with the dyeing process listed in the Colour Index, including several structural varieties of dyes, such as acidic, reactive, basic, disperse, azo, diazo, anthraquinone-based and metal-complex dyes [3]. The removal of colour from wastewaters is often more important than the removal of the soluble colourless organic substances, which usually contribute to the major fraction of the biochemical oxygen demand (BOD). Methods for the removal of BOD from most effluents are fairly well established; dyes, however, are more difficult to treat because their synthetic origin are mainly complex aromatic molecular structures, often synthesized to resist fading on exposure to sweat, soap, water, light or oxidizing agents [1,6]. This renders them more stable and less amenable to biodegradation [7,8].

Many approaches, including physical and/or chemical processes, have been used in the treatment of industrial wastewater containing dye but such methods are often very costly and not environmentally safe [9,10]. Methods utilizing powdered activated carbon and activated bentonites have been commonly used [11,12]. However, the large amount of sludge generated and the low efficiency of treatment with respect to some dyes have limited their use [13]. Colour removal using ozone is also usually effective and fairly rapid, but not all the methods employed give satisfactory results especially for some dispersed dyes [1]. Another widely used treatment method for coloured effluents is the physical-chemical flocculation with metal hydroxides assisted by polymer flocculants [14], while the application of pre-mixed polyelectrolyte complexes made by the interaction of aqueous solutions of polycation and polyanionis accepted as a more practical method [15]. Such complex particles are able to bind disperse dyes effectively over large distances due to their size and structure via hydrophobic as well as electrostatic interaction forces [16]. However, because dye molecules or their aggregates are incomparably smaller than such inorganic particles, and in some cases also uncharged, it is necessary to apply other flocculation principles. 

Interest in the pollution potential of textile dyes has been primarily prompted by concern over their possible toxicity and carcinogenicity. This is mainly because many dyes are made from known carcinogens, such as benzidine and other aromatic compounds, all of which might be transformed because of microbial metabolism [6]. It has also been shown that azo- and nitro- compounds are reduced in sediments [17] and in the intestinal environment [18], resulting in the regeneration of the parent toxic amines. Some disperse dyes have also been shown to bio-accumulate [19], while heavy-metal ions from textile effluents have also been reported at high concentrations in both algae and higher plants exposed to such effluents [20]. In recent times, industries have been faced with more stringent effluent treatment regulations and are required to lower the colour content in their wastewater before discharge into the surface water [21]. This means that for most textile industries, developing on-site or in-plant facilities to treat their own effluents before discharge is fast approaching actuality. New flocculation mechanisms are therefore attracting more attention [22].

The removal of dyes from wastewater presents a formidable challenge, as most dyes are completely soluble in aqueous solutions [23]. Although dyes constitute only a small portion of the total volume of waste discharge in textile processing, these compounds are not readily removed by typical microbial-based waste-treatment processes [24]. Furthermore, dyes can be detrimental to the microbial population present in such treatment works and may lead to decreased efficiency or treatment failure in such plants [25]. Bioflocculants can be used in various industries such as in wastewater treatment, domestic, brewage, and pharmaceutical wastewater treatment, textile industry, dredging/downstream processes and in food-processing and fermentation industries [26,27]. Recently, Zhang *et al. * [28] reported that bioflocculants produced by bacterial strains xn11 + xn7 were effective in decolorizing basic fuchsin (100 mg L^−1^) but less effective in decolorizing reactive black (50 mg L^−1^), exhibiting a decolorization efficiency of 93 and 35%, respectively. Biological methods, being relatively cheap and simple to use, have been the focus of recent studies on dye degradation and decolourization [18]. Therefore, the objective of this study was to evaluate the ability of the bioflocculants, produced by bacteria indigenous to a wastewater treatment plant in South Africa, to remove dyes from textile industrial effluents.

## 2. Results

### 2.1. Dye Removal by the Bacterial Bioflocculants

**Figure 1:**
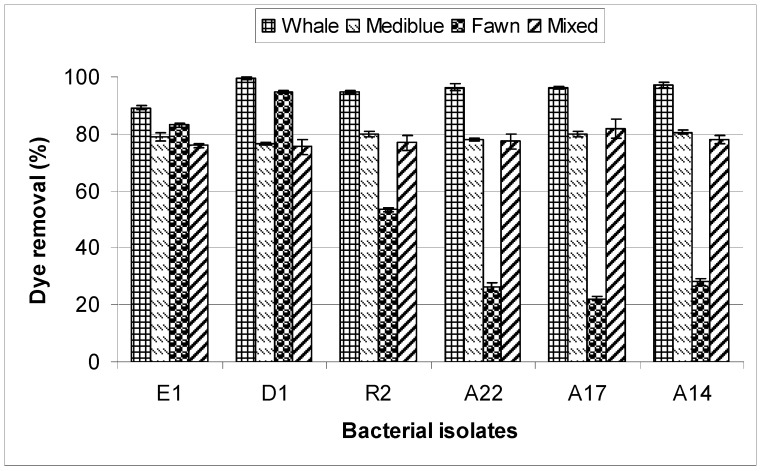
Dye removal from the textile industrial effluents using bioflocculants produced by the bacterial isolates at 10 ppm concentration.

Analysis of 16S rRNA gene sequence identified the isolates as *Bacillus subtilis* (E1), *Exiguobacterium acetylicum* (D1), *Klebsiella terrigena* (R2), *Staphylococcus aureus* (A22), *Pseudomonas pseudoalcaligenes* (A17), and *Pseudomonas plecoglossicida* (A14). Bioflocculants produced by all the bacterial isolates were able to decolourize the textile industrial effluent with a removal rate of up to 97.04%, 80.61%, 94.93% and 81.64% for whale, mediblue, fawn and mixed dye, respectively (Figure 1). A very low removal of fawn dye was observed for isolates A14, A17 and A22, with only 22.13–28.18% removal. Overall, whale dye was most effectively removed from the industrial textile dye effluent, with a removal efficiency ranging from 89.15–97.04% for all the bioflocculants. The chemical properties of the textile dyes used in this study are shown in Table 1.

### 2.2. Effect of pH and Temperature on Dye Removal

**Figure 2:**
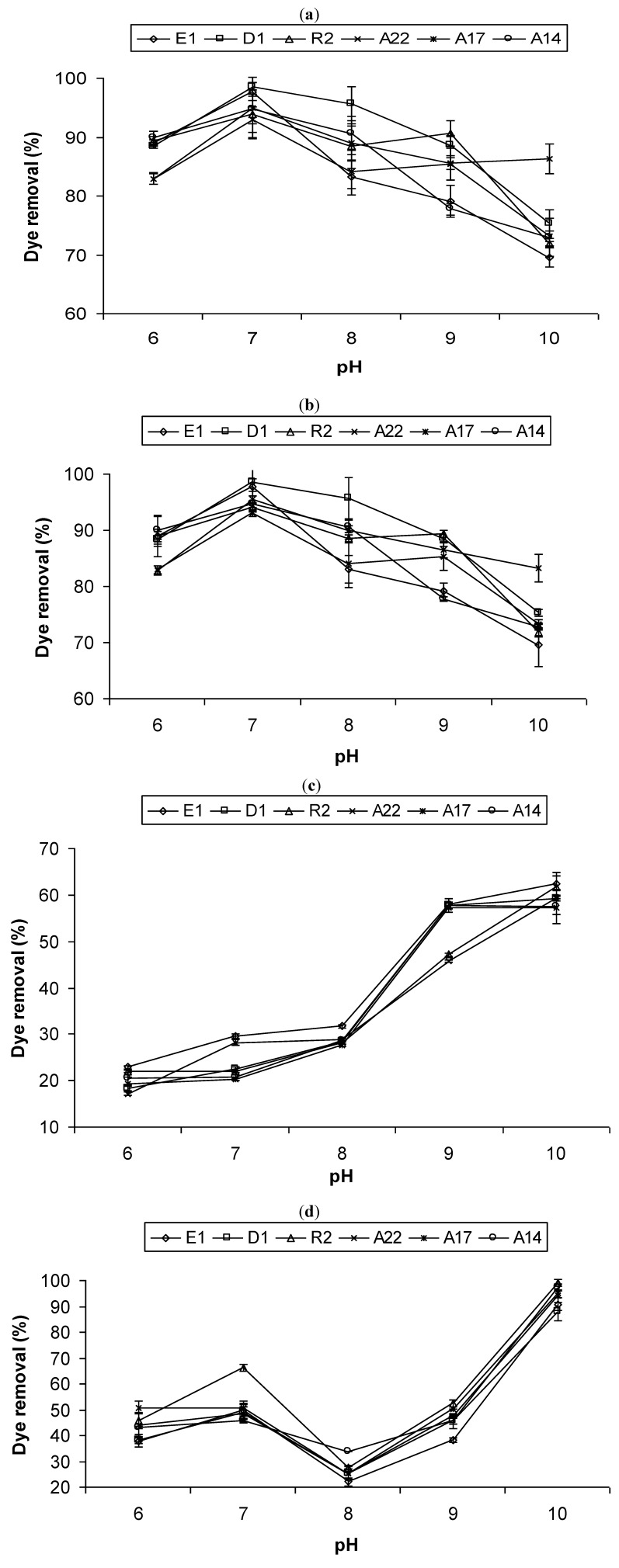
Effects of pH on the removal of (**a**) whale dye; (**b**) mediblue dye; (**c**) fawn dye; and (**d**) mixed dye from textile industrial effluents.

**Figure 3:**
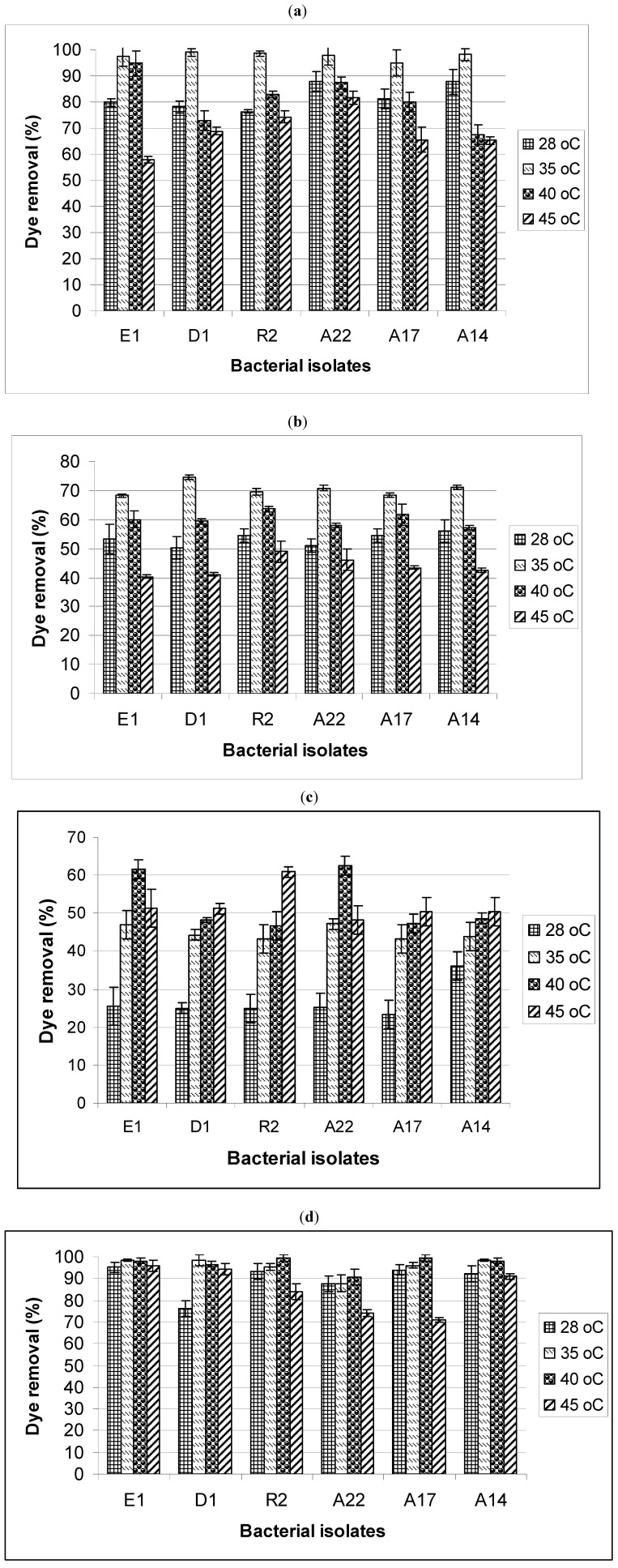
Effects of temperature on the removal of (**a**) whale dye; (**b**) mediblue dye; (**c**) fawn dye; and (**d**) mixed dye from textile industrial effluents.

The effect of pH and temperature on the removal of the dyes by the bacterial bioflocculants is shown in Figure 2 and Figure 3, respectively. A pH of 7 was found to be optimum for the removal of both whale (Figure 2a) and mediblue (Figure 2b) dyes for all the bacterial bioflocculants, with a slight decrease in removal rate at pHs 6, 8, and 9. At pH 10, up to 28.15% reduction in the whale and mediblue dye removal efficiency was observed for isolate E1, compared to the removal rate at the optimum pH. For fawn (Figure 2c) and mixed dye (Figure 2d), the highest removal was observed at pH of 10, except for bioflocculant from isolates A14 and A17 with an optimum pH of 9 for fawn dye removal (Figure 2c). There was an increase in the removal rate of fawn dye with an increase in pH. Similarly, the optimum temperature for whale dye (Figure 3a) and mediblue dye (Figure 3b) removal was 35 °C for all the bacterial bioflocculants, while those for fawn and mixed dye was found to vary between 40 –45 °C (Figure 3c) and 35–40 °C (Figure 3d), respectively.

**Table 1:** Chemical properties of the textile dyes used in this study.

Colour	Classification	Dyes	Concentration (%)	Bulk Density (kg/m^3^)
Whale	Azo	Dianix yellow S-6G	0.37	≈600
		Dianix rubine S-3B	0.085	
		Dianix navy CC	1.70	
Medi-blue	Anthraquinone	Avolan 15 LIQ	0.30	400–600
		Dianix turquois blue S-BG	0.35	
		Dianix blue KFBL	0.0084	
Fawn	Azo	Dianix yellow S-6G	0.048	450–520
		Tiacron/rubine – C-BT 200	0.038	
		Dianix blue K-FBL	0.018	

### 2.3. Dye Removal in the Presence of Cationic Salts

The effect of cations on the removal of the dyes is depicted in Table 2. The most effective cation for the removal of whale dye was MnCl_2_, followed by MgSO_4_ and CaCl_2_, while CTAB was the least, with as low as 28.41% dye removal observed using bioflocculant produced by isolate R2. The most effective cation for the removal of mediblue dye was MnCl_2_ for all the bioflocculants, followed by MgSO_4_, CTAB and CaCl_2_, with up to 99.58% removal observed for bioflocculant from isolate E1 in the presence of MnCl_2_. The least mediblue dye removal rate of 49.39% was observed with bioflocculant from isolate D1. Fawn dye seems to be better removed in the presence of CTAB for bioflocculant from all isolates, except for isolate A14, where only 46.83% removal was obtained. Generally, low removal efficiency was obtained with the mixed dye in the presence of CTAB, MgSO_4_, and CaCl_2_. However, removal rate as high as 99.11% was observed in the presence of MnCl_2_ using bioflocculant from isolate A17.

**Table 2:** Effects of cations ^a^ on the removal of dyes from the wastewater effluents by the bacterial bioflocculants at 10 ppm concentration.

	Dye removal (%)																		
Isolate	CTAB					CaCl_2_					MnCl_2_					MgSO_4_			
	WD	MD	FD	MD		WD	MD	FD	MD		WD	MD	FD	MD		WD	MD	FD	MD
E1	36.58	72.87	96.83	59.85		68.36	50.34	53.47	78.47		99.10	99.58	77.40	83.74		99.57	77.70	71.03	62.33
D1	61.60	72.39	98.29	40.92		75.36	49.39	50.21	65.07		98.20	84.03	80.23	76.85		94.89	70.63	73.62	56.28
R2	28.41	76.66	89.27	40.41		73.82	53.98	57.00	75.60		98.20	94.12	76.04	98.52		97.45	85.87	74.17	68.37
A22	29.19	66.56	88.54	43.48		77.46	49.93	53.32	64.12		99.10	87.82	73.82	95.07		88.51	79.55	74.17	61.40
A17	37.71	83.82	74.88	20.97		74.91	52.50	99.01	78.47		99.10	94.96	76.65	99.11		93.62	81.78	70.30	59.54
A14	38.92	77.04	46.83	35.29		74.55	56.14	99.43	84.21		97.21	95.80	83.24	99.02		95.32	89.22	68.08	52.56

WD = Whale Dye; MD = Mediblue Dye; FD = Fawn Dye; MD = Mixed Dye; ^a^ 100 μL of the 1M solution of the different cationic salts was added to a mixture of the dye effluent and bioflocculant in a test tube before determining the flocculating activities of the bacterial bioflocculants and the dye removal rate.

## 3. Discussion

In this study, the dye-removal potential of bioflocculants produced by bacterial isolates indigenous to a wastewater treatment plant was investigated. Bioflocculants from all the isolates were able to remove the different dyes tested from industrial wastewater to varying degrees, possibly by causing aggregation of particles and cells due to bridging and charge neutralization as previously reported [29]. Bridging occurs when the flocculant extends from the particle’s surface into the solution for a distance greater than the distance over which the interparticle repulsion acts. In this case, the biopolymer can adsorb to other particles to form flocs [30,31]. The results obtained in this study demonstrate that the removal of all the dyes tested was directly proportional to the bioflocculant concentration until an optimum concentration was reached (results not shown). Shubo *et al.* [32] also reported that higher decolourization of dyes can be achieved by increasing the concentration of the bioflocculants. In order to be effective in destabilization, a polymer molecule must contain chemical groups that can interact with sites on the surface of the colloidal particle. A particle-polymer-particle complex is thus formed in which the polymer serves as a bridge. If a second particle is not available in time, the extended segments may eventually adsorb on other sites on the original particle, so that the polymer is no longer capable of serving as a bridge [29,33]. A direct stoichiometric relationship between optimum polymer dosage and colloid concentration has previously been observed [34].

The optimum pH was observed at pH 7 for whale and medi-blue dyes removal (Figure 2a,b), while for fawn and mixed dyes it was observed at pH 10 (Figure 2c,d). According to Willmott [35], the optimum pH for colour removal is often at a neutral or a slightly alkaline pH and decolorization tends to decrease rapidly at strongly acidic or strongly alkaline pH values. As a result, the coloured wastewater is often buffered to enhance the colour removal performance. Colour removal in the alkaline pH range is presumably due to adsorption onto hydroxide flocs. Biological reduction of the azo bond of whale and fawn dye can result in an increase in pH due to the formation of aromatic amine metabolites that are more basic than the original azo compound [35]. Chang *et al.* [36] found that the dye reduction rate increased nearly 2.5-fold as the pH was raised from 5.0 to 7.0, with dye removal efficiency being insensitive to pH in the range of 7.0–9.5. These findings are in accordance with the results obtained in this study. On the other hand, Mittal and Gupta [37] observed a decrease in the removal of three cationic dyes, viz., Orlamar Red BG, Orlamar Blue G and Orlamar Red GTL, with decreasing pH, using the bioflocculant of the fungus, *Fomitopsis carnea. *This was attributed to the fact that repulsive forces between coloured dye cations in solution and biosorbent surface charged positively at pH values lower than 3.0. In many systems, decolorization increases with increasing temperature, within a defined range that depends on the system [36]. In this study, the temperature required to produce maximum decolorization was found to range between 35–45 °C for all the textile dyes tested (Figure 3). These results are consistent with those reported by Pearce *et al.* [13]. Also, an optimum adsorption temperature of 35 °C was reported for Remazol Black B reactive dye using the bioflocculant from *Rhizopus arrhizus*, with a decreased adsorption observed with further increase in temperature due to decreased surface activity [38]. The decline in colour removal activity at higher temperatures has been attributed to the possible loss of cell viability [13]. 

The addition of divalent cations such as Mn^2+^, Mg^2+^, and Ca^2+^ enhanced both the flocculating activity and the decolourization of the dyes (Table 2). Cations stimulate flocculating activity by neutralizing and stabilizing the residual negative charge of functional groups and by forming bridges between particles. Divalent and trivalent cations increase the initial adsorption of biopolymers on suspended particles by decreasing the negative charge on both the polymer and the particle [31]. Divalent cations, viz., Mn^2+^, Mg^2+^, and Ca^2+^ have been shown to bind to the bioflocculants to form complexes, thereby stimulating flocculation and decolourization [39]. However, Fujita *et al.* [40] and Yim *et al.* [41] indicated that the flocculating activity of *Citrobacter *sp. TKF04 and *Gyrodinium impudicum* KG03 was not enhanced by the addition of any cations, including Ca^2+^. Dyeing processes consume large amounts of salts, thus making salt concentration in dye-containing wastewater one of the important factors that influence biosorption capacity [42,43]. Textile wastewaters may include metal ions beside dyes and salts due to metal-containing dyes used in textile industry. Metal ions may therefore play an important role in influencing biosorption rate and capacity as they might compete with dye molecules for the binding sites or stimulate the biosorption of dye onto biomass [43]. 

Among the four dyes used in this study, whale dye was most easily decolourized, followed by medi-blue, mixed dyes and fawn dye, respectively. One explanation for this, according to Pearce *et al.* [13], could be the acidic nature of the fawn dye. Acid dyes are the most problematic and difficult to decolourize due to their inert chemical structure and the attached phenyl, methyl, methoxy, nitro and sulphonate groups. Also, Sanghi *et al.* [44] suggested that removal of colour from dye solutions is complex and may be due to physico-chemical processess of coagulation and/or chelation–complexation type reactions. These techniques decolorize textile wastewater by partially decomposing the dye molecules and thereafter leaving the harmful residues in the effluent [45,46]. Fang *et al.* [45] reported that the removal of dyes (anionic azo-dyes) by flocculants may be attributed to both charge neutralization and bridging effect, with the former being the dominant mechanism. The dye structure appears to be conducive to chelation/complex formation reactions with coagulants leading to the formation of insoluble metal dye complexes that may precipitate from solution. In the present study, colour removal could probably be due to aggregation or precipitation and adsorption of colouring substances onto the coagulant species. 

## 4. Experimental

### 4.1. Bioflocculant-Producing Bacterial Isolation and Identification

Bioflocculant-producing bacteria were isolated from the activated sludge sample collected from the Northern Wastewater Treatment Plant in Durban, South Africa. Identification was done using standard biochemical tests, the API test kit (Biomerieux, Marcy l'Etoile, France) as well as the 16S rRNA gene sequence analysis as described elsewhere [47]. Total genomic DNA was isolated from LB-grown cells using QIAamp DNA Miniprep Kit (Qiagen, Hilden, Germany) following manufacturer’s instructions, and used directly as the template for PCR amplification. The 16S rDNA of the bacterial isolates were amplified with the oligonucleotide primers: 63f and 1387r described by Marchesi *et al.* [48]. 

### 4.2. Bioflocculant Production

Bacteria were cultivated in 250 mL Erlenmeyer flasks containing 30 mL YMPG medium for 20 h at 28 °C on a rotary shaker at 220 rpm. A 0.7 mL aliquot was added into a 500 mL Erlenmeyer flask containing 70 mL of production medium (0.5% yeast extract, 0.5% polypeptone, 2% ethanol, 1% glycerol, 0.05% K_2_HPO_4_, 0.05% MgSO_4_.7H_2_O, 0.2% NaCl, and 0.2% CaCO_3_). The flasks were incubated for 3 days at 28 °C before determining the amount of bioflocculant produced by the ethanol precipitation method [49].

### 4.3. Textile Dyes Used in This Study

The three types of dispersible dyes selected for use in this study were whale, medi-blue, fawn and a mixture of dyes. The dyes are anionic and their chemical properties are shown in Table 1. The dyes were collected from a textile industry in Hammarsdale (KwaZulu-Natal, South Africa) directly from the large storage tanks immediately after cooling of the dyes. Mixed dye was collected from the textile industrial treatment plant (also in Hammarsdale) and composed of a variety of dyes from all the textile industries around Hammarsdale. 

### 4.4. Dye Removal by the Bacterial Bioflocculants

In a test tube, undiluted dye effluent (9 mL) was mixed with different concentrations of the bacterial bioflocculants (1 mL, 2–10 ppm). The components of the test tube were mixed using a Labcon shaker at 200 rpm for 1 min, and then at 60 rpm for another 5 min. The dyes were left to settle for 60 min and the OD was measured spectrophotometrically at 550 nm using a UV/Visible spectrophotometer, Ultrospec 2000 (Pharmacia Biotech, Piscataway, NJ, USA). The decolourization efficiency or the percentage removal of dyes was calculated using the Equation (1): Where: C0 is the absorbance of the untreated dye and C is the absorbance after the treatment with bacterial bioflocculants [32].

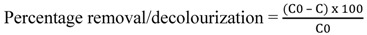
(1)
where C0 is the absorbance of the untreated dye and C is the absorbance after the treatment with bacterial bioflocculants [32].

### 4.5. Effect of pH and Temperature on Dye Removal

To determine the effect of pH on dye removal, the initial pH of dye-containing wastewater was adjusted accordingly in the test tubes (pH range of 6–10) using 2 N HCl or NaOH. To determine the effect of temperature, the test tubes containing dye wastewater were incubated at different temperatures (28 °C, 35 °C, 40 °C and 45 °C). The decolourization efficiency or the percentage removal of dyes was determined as previously described.

### 4.6. Determination of the Effects of Cationic Salts on Dye Removal

Different cationic compounds (CaCl_2_·2H_2_O, MgSO_4_·7H_2_O, MnCl_2_·7H_2_O and CTAB) were used to determine the effect of salts on dye removal by the bacterial bioflocculants at the optimum temperature and pH. A 100 µL of 1M solution of each cation was added to the mixture of dye effluent and bacterial bioflocculant before determining the decolourization efficiency or the percentage removal of dyes as previously described.

## 5. Conclusions

Results obtained in this study suggest that bacterial bioflocculants may provide a promising economical and cleaner alternative to replace or supplement current treatment processes for the removal of very high concentrations of dyes in industrial wastewater effluents, as they are biodegradable and easily available and sustainable. However, the decolourization efficiency by the bacterial bioflocculants depends largely on the type of dye, pH, temperature and flocculant concentration. 

## References

[1] Aksu Z. (2005). Application of biosorption for the removal of organic pollutants: A review. Process Biochem..

[2] Pagga U., Brown D. (1986). The degradation of dyestuffs. Chemosphere.

[3] Banat I.M., Nigam P., Singh D., Marchant R. (1996). Microbial decolourization of textile-dye-containing effluents. Bioresour. Technol..

[4] Crini G. (2006). Non-conventional low-cost adsorbents for dye removal. Bioresour. Technol..

[5] Dos Santos A.B., Cervantes F.J., van Lier J.B. (2007). Review paper on current technologies for decolourization of textile wastewaters: Perspectives for anaerobic biotechnology. Bioresour. Technol..

[6] Khan A.A., Husain Q. (2007). Decolorization and removal of textile and non-textile dyes from polluted wastewater and dyeing effluent by using potato (*Solanum tuberosum*) soluble and immobilized polyphenol oxidase. Bioresour. Technol..

[7] Fewson C.A. (1988). Biodegradation of xenobiotic and other persistent compounds: the causes of recalcitrance. Trends Biotechnol..

[8] Seshadri S., Bishop P.L., Agha A.M. (1994). Anaerobic/aerobic treatment of selected azo dyes in wastewater. Waste Manag..

[9] Nigam P., Banat I.P., Singh D., Marchant R. (1996). Microbial process for the decolourization of textile effluent containing azo, diazo, and reactive dyes. Process Biochem..

[10] Rauf M.A., Shehadi I.A., Hassan W.W. (2007). Studies on the removal of neutral red on sand from aqueous solution and its kinetic behaviour. Dyes Pig..

[11] Pala A., Tokat E. (2002). Colour removal from cotton textile industry wastewater in an activated sludge system with various additives. Water Resour..

[12] Yavuz O., Aydin A.H. (2002). The removal of acid dye from aqueous solution by different adsorbents. Fresenius Environ. Bull..

[13] Pearce C.I., Lloyd J.R., Guthrie J.T. (2003). The removal of colour from textile wastewater using whole bacterial cells. Dyes Pig..

[14] Choy J.H., Shin W.S., Lee S.H., Joo D.J., Lee J.D., Choi S.J. (2001). Application of synthetic poly (DADM) flocculants for dye wastewater treatment. Sci. Technol..

[15] Petzold G., Nebel A., Buchhammer H.M., Lunkwitz K. (1998). Preparation and characterization of different polyelectrolyte complexes and their application as flocculants. Colloid Polym. Sci..

[16] Buchhammer H.M., Oelmann M., Petzold G. (2001). Flocculation of disperse dyes in effluents with polyelectrolyte complexes. Melliand Int..

[17] Weber E.J., Wolfe N.L. (1987). Kinetics studies of reduction of aromatic azo compounds in anaerobic sediment/water systems. Environ. Toxicol. Chem..

[18] Sirianuntapiboon S., Srisornsak P. (2007). Removal of disperse dyes from textile wastewater using bio-sludge. Bioresour. Technol..

[19] Baughman G.L., Perenich T.A. (1988). Fate of dyes in aquatic systems: I. Solubility and partitioning of some hydrophobic dyes and related compounds. Environ. Toxicol. Chem..

[20] Srivastava P.N., Prakash A. (1991). Bioaccumulation of heavy metals by algae and wheat plants fed by textile effluents. J. Water Pollut. Control Fed..

[21] Pinheiro H.M., Touranud E., Thomas O. (2004). Aromatic amines from azo dye reduction: status review with emphasis on direct UV spectrophotometric detection in textile industry wastewaters. Dyes Pigm..

[22] Petzold G., Mende M., Lunkwitz K., Schwarz S., Buchhammer H.M. (2003). Higher efficiency in the flocculation of clay suspensions by using combinations of oppositely charged polyelectrolytes. Colloid. Surf. A.

[23] Sanayei Y., Ismail N., Teng T.T., Morad N. (2010). Studies on flocculating activity of bioflocculant from closed drainage system (CDS) and its application in reactive dye removal. Int. J. Chem..

[24] Li J., Li M., Li J., Sun H. (2007). Decolourization of azo dye direct scarlet 4BS solution using exfoliated graphite under ultrasonic irradiation. Ultrason. Sonochem..

[25] Ogawa T., Shibata M., Yatome C., Idaka E. (1988). Growth inhibition of *Bacillus subtilis* by basic dyes. Bull Environ. Contam. Toxicol..

[26] Wang S.G., Gong W.X., Liu X.W., Tian L., Yue Q.Y., Gao B.Y. (2007). Production of a novel bioflocculant by culture of *Klebsiella mobilis* using dairy wastewater. Biochem. Eng. J..

[27] Gong W.-X., Wang S.-G., Sun X.-F., Liu X.-W., Yue Q.-Y., Gao B.-Y. (2008). Bioflocculant production by culture of *Serratia ficaria* and its application in wastewater treatment. Bioresour. Technol..

[28] Zhang C.L., Cui Y., Wang Y. (2012). Bioflocculant produced from bacteria for decolorization, Cr removal and swine wastewater application. Sustain. Environ. Res..

[29] Salehizadeh H., Shojaosadati S.A. (2001). Extracellular biopolymeric flocculants: recent trends and biotechnological importance. Biotechnol. Adv..

[30] Hantula J., Bamford D.H. (1991). The efficiency of the protein dependent flocculation of *Flavobacterium* sp. Appl. Microbiol. Biotechnol..

[31] Levy N., Magdasi S., Bar-Or Y. (1992). Physico-chemical aspects in flocculation of bentonite suspensions by a cyanobacterial bioflocculant. Water Res..

[32] Shubo D., Yu G., Yen Peng T. (2005). Production of a bioflocculant by *Aspergillus parasiticus* and its application in dye removal. Colloids Surf. B.

[33] Salehizadeh H., Shojaosadati S.A. (2002). Isolation and characterization of the bioflocculant produced by *Bacillus firmus*. Biotechnol. Lett..

[34] Mishra A., Bajpai M. (2005). Flocculation behaviour of model textile wastewater treated with a food grade polysaccharide. J. Hazard Mat..

[35] Willmott N.J. (1997). The use of bacteria-polymer composites for the removal of colour from reactive dye effluents. Ph.D. thesis.

[36] Chang J.S., Chou C., Lin Y.C., Lin P.J., Ho J.Y., Hu T.L. (2001). Kinetic characteristics of bacterial azo-dye decolourization by *Pseudomonas luteola*. Water Res..

[37] Mittal A.K., Gupta S.K. (1996). Biosorption of cationic dyes by dead macro-fungus *Fomitopsis carnea*: batch studies. Water Sci. Technol..

[38] Aksu Z., Tezer S. (2006). Equilibrium and kinetic modelling of biosorption of *Remazol Black* B by *Rhizopus arrhizus* in a batch system: effect of temperature. Process Biochem..

[39] Kurane R., Hatamochi K., Kakuno T., Kiyohara M., Kawaguchi K., Mizuno Y., Hirano M., Taniguchi Y. Purification and characterization of lipid bioflocculant produced* Rhodococcus erythropolis*. Biosci. Biotechnol. Biochem..

[40] Fujita M., Ike M., Tachibana S., Kitada G., Kim S.M., Inoue Z. (2000). Characterization of a bioflocculant produced by *Citrobacter *sp. TKF04 from acetic and propionic acids. J. Biosci. Bioeng..

[41] Yim J.H., Kim S.J., Ahn S.H., Lee H.K. (2007). Characterization of a novel bioflocculant, p-KG03, from a marine dinoflagellate, *Gyrodinium impudicum* KG03. Bioresour. Technol..

[42] Zhou J.L., Banks C.J. (1991). Removal of humic acid fraction by *Rhizopus arrhizus*: Uptake and kinetic studies. Environ. Technol..

[43] Zhou J.L., Banks C.J. (1993). Mechanism of humic acid colour removal from natural waters by fungal biomass biosorption. Chemosphere.

[44] Sanghi R., Bhattacharya B., Dixit A., Singh V. (2006). *Ipomoea dasysperma* seed gum: An effective natural coagulant for the decolourization of textile dye solutions. J. Environ. Manag..

[45] Fang R., Cheng X., Xu X. (2010). Synthesis of lignin-base cationic flocculant and its application in removing anionic azo-dyes from simulated wastewater. Bioresour. Technol..

[46] Qin L., Zhang G., Meng Q., Xu L., Lv B. (2012). Enhanced MBR by internal micro-electrolysis for degradation of anthraquinone dye wastewater. J. Chem. Eng..

[47] Olaniran A.O., Pillay D., Pillay B. (2008). Aerobic biodegradation of dichloroethenes by indigenous bacteria isolated from contaminated sites in Africa. Chemosphere.

[48] Marchesi J.R., Sato T., Weightman A.J., Martin T.A., Fry J.C., Hiom S.J., Dymock D., Wade W.G. (1998). Design and evaluation of useful bacterium-specific PCR primers that amplify genes coding for 16S rRNA. Appl. Environ. Microbiol..

[49] Kurane R., Hatamochi K., Kakuno T., Kiyohara M., Kawaguchi K., Mizuno Y., Hirano M., Taniguchi Y. (1994). Production of a bioflocculant by *Rhodococcus erythropolis* S-l grown on alcohols. Biosci. Biotechnol. Biochem..

